# A New Name for *Pneumocystis* from Humans and New Perspectives on the Host-Pathogen Relationship

**DOI:** 10.3201/eid0809.020096

**Published:** 2002-09

**Authors:** James R. Stringer, Charles B. Beard, Robert F. Miller, Ann E. Wakefield

**Affiliations:** * University of Cincinnati, Cincinnati, Ohio, USA; † Centers for Disease Control and Prevention, Atlanta, Georgia, USA; ‡ University College London, United Kingdom; § University of Oxford, UK

**Keywords:** P*neumocystis*, nomenclature, human, epidemiology, genetics

## Abstract

The disease known as *Pneumocystis carinii* pneumonia (PCP) is a major cause of illness and death in persons with impaired immune systems. While the genus *Pneumocystis* has been known to science for nearly a century, understanding of its members remained rudimentary until DNA analysis showed its extensive diversity. *Pneumocystis* organisms from different host species have very different DNA sequences, indicating multiple species. In recognition of its genetic and functional distinctness, the organism that causes human PCP is now named *Pneumocystis jiroveci* Frenkel 1999. Changing the organism’s name does not preclude the use of the acronym PCP because it can be read “*Pneumocystis* pneumonia.” DNA varies in samples of *P. jiroveci,* a feature that allows reexamination of the relationships between host and pathogen. Instead of lifelong latency, transient colonization may be the rule.

## Clinical Importance of *Pneumocystis*

The disease known as *Pneumocystis carinii* pneumonia (PCP) is one of the leading causes of illness and death in persons with impaired immunity. The disease has been described in immunocompromised patients for many years, including outbreaks in malnourished young children in orphanages in Iran in the 1950s (1–6). The AIDS epidemic, however, marked the beginning of the disease’s impact on a substantial number of patients. PCP has long been the most common serious AIDS-defining opportunistic infection in the United States. The introduction of highly active antiretroviral therapy (HAART) for the treatment of HIV infection has been accompanied by substantial reductions in mortality and the incidence of opportunistic infections, including PCP (7). Despite these advances, *Pneumocystis* remains a major pathogen in HIV-infected persons who either are not receiving or are not responding to HAART and among those who are unaware of their HIV status. PCP is also of clinical importance in people immunocompromised for reasons other than HIV, such as organ transplantation or chemotherapy for malignant diseases (8). In addition, *Pneumocystis* infection has been documented recently in persons who are mildly immunocompromised, including those with chronic lung disease (9).

### Need for a Change in Nomenclature

*Pneumocystis* organisms were first reported by Chagas in 1909 (10), but he mistook them for a morphologic form of *Trypanosoma cruzi*. Within a few years of this first report, further studies established that the microbe in question was not a trypanosome but a new species altogether, named *Pneumocystis carinii* (11).

From the time of its discovery, until late in the 1980s, *Pneumocystis* was widely thought to be a protozoan. These views were based on several criteria: 1) strong similarities in microbe morphology and host pathology, 2) absence of some phenotypic features typical of fungi, 3) presence of morphologic features typical of protozoa, 4) ineffectiveness of antifungal drugs, and 5) effectiveness of drugs generally used to treat protozoan infections. Some investigators pointed out that *Pneumocystis* organisms exhibit morphologic similarities to fungi (2). Nevertheless, the protozoan hypothesis remained predominant until 1988, when DNA analysis demonstrated that *Pneumocystis* is a fungus, albeit an odd one, lacking in ergosterol and very difficult to grow in culture (12,13).

Soon after the proper classification of *Pneumocystis* had been determined at the kingdom level, additional DNA data showed that *Pneumocystis* organisms in different mammals are quite different. These data led to interim name changes (14), but it was not until 1999 that the first valid new binomial appeared. The organism that causes human PCP is now named *Pneumocystis jiroveci* Frenkel 1999 (pronounced “yee row vet zee”), in honor of the Czech parasitologist Otto Jirovec, who is credited with describing the microbe in humans (15). The primary purpose of this article is to explain what led to the name change and why the new name is necessary, useful, and workable for all concerned. For a more extensive review of the systematics and nomenclature of *Pneumocystis*, see Stringer’s review of workshops on the subject (16). The DNA sequence information that led to the renaming of *Pneumocytsis* organisms also provided the tools needed to better understand the relationships between these microbes and the hosts they inhabit. Thus, the secondary purpose of this article is to summarize data on these relationships, focusing on current views on the relationship between *P. jiroveci* and humans.

## Complexity of the Genus

One reason that a definitive nomenclature has been slow to develop is that *Pneumocystis* organisms have been difficult to study. Attempts to develop an in vitro culture system have had limited success. Cultivation of *Pneumocystis* organisms in vitro requires a large seed population and supports rather modest increases in organism number for a very limited period of time (17). An exception to the rule was recently reported (18); however, this method has not been established in other laboratories. The fastidiousness of *Pneumocystis* organisms greatly hampered early efforts to understand them. Fortunately, advances in DNA analysis technology allowed progress in the absence of a robust culture system.

### *Pneumocystis jiroveci* as a Distinct Species

Phenotypic differences between *P. jiroveci* and other species of *Pneumocystis* were noted decades ago (19). More recent descriptions echo these reports (20). On the basis of phenotypes, Frenkel first proposed the name *Pneumocystis jiroveci* in 1976. The name was not validly published, however, under the then-prevailing specifications of the International Code of Zoological Nomenclature. Thus, the name did not gain acceptance at that time.

The first indication of a molecular difference between *P*. *jiroveci* and *Pneumocystis* from laboratory animals came from analyses of protein sizes (21,22). However, the importance of these differences was difficult to judge because the *Pneumocystis* was prepared directly from the lung of the host, leaving open the possibility that differences could have been due to extrinsic factors such as contamination with host proteins, host-mediated modification of *Pneumocystis* proteins, or presence of dead *Pneumocystis* organisms.

DNA analysis provided the information needed to clarify the issue and to establish that the organisms from humans and other animals are quite different (23). The most powerful approach has been to use polymerase chain reaction (PCR). Wakefield developed primers that amplify DNA from all known species of *Pneumocystis* (24,25). When these primers have been used on human-derived samples of *Pneumocystis*, the only DNA found has been that of *P. jiroveci.* Moreover, *P. jiroveci* DNA has not been found in lung samples from any other mammals, including nonhuman primates (26). The PCR data are supported by the results of sequencing cloned genes. Several genes or gene fragments have been cloned from human-derived *Pneumocystis* (27–30). In all cases, the gene sequence is very different from its orthologues in *Pneumocystis* organisms from other host species. Genetic divergence data also argue that *P. jiroveci* is a distinct species. The 18S rRNA sequences from *P. jiroveci* (i.e., human-derived) and *P. carinii* (i.e., rat-derived) differ by 5%. This level of divergence is comparable with that between *Pneumocystis* organisms and *Taphrina deformans* (a plant fungal pathogen), whose 18S rRNA sequences differ by approximately 6%. In contrast, species in the genus *Saccharomyces* can differ by as little as 1% at the 18S rRNA locus.

The genetic divergence between *P. jiroveci* and other *Pneumocystis* organisms is typical of the genus. When *Pneumocystis* from different host species are compared by DNA sequence analysis, they always differ (23,25,31–33). In addition, experiments with rats, mice, ferrets, and monkeys have demonstrated host-species specificity (34–36). For example, when *Pneumocystis* organisms were taken from a rat and transferred to a mouse, proliferation was not evident, and no disease resulted (34). In contrast, when *Pneumocystis* organisms from a rat were transferred to another rat, they proliferated to a very high number and caused severe disease. Transfer experiments that seem to show lack of specificity have been reported, but these reports did not show that the proliferating organisms were the same species of *Pneumocystis* as those introduced, leaving open the possibility that endogenous organisms were responsible for the infection.

*Pneumocystis* organisms might be obligate parasites that have evolved to survive in a particular host species. Co-evolution of parasite and host might be expected in such a case. Note, in this regard, that *P. jiroveci* is most similar to organisms isolated from other primates (37). This finding fits with the obligate parasite conjecture. However, the host specificity data also fit with an alternative scenario: there could be many free-living species of *Pneumocystis*, one of which is capable of invading humans, others of which are capable of invading nonhuman primates, and the like. In this scenario, the similarity between *P. jiroveci* and the *Pneumocystis* organisms found in nonhuman primates would reflect the similarities between humans and other primates. If *P. jiroveci* is not an obligate parasite, finding it outside the human body should be possible. *P. jiroveci* DNA has been detected in samples of airborne fungal spores (24) and in a sample of pond water (38). However, the number of *P. jiroveci* in the environment seems to be very low, leaving open the possibility that these “free forms” of the organism may have been deposited by humans. *P. jiroveci* could be an obligate parasite, spores of which can survive in the environment long enough to infect a new host, should one be encountered. Resolving this question awaits the availability of a system capable of detecting infectious *Pneumocystis* organisms in the air, water, or soil.

Soon after DNA sequence data began to appear, name changes were suggested (14,39). However, naming new species seemed premature to many because of concerns about the possibility of creating false species by misinterpreting the importance of a limited amount of DNA sequence data. Consequently, a provisional trinomial nomenclature was adopted. This system referred to the different kinds of *Pneumocystis* organisms as special forms of *P. carinii* Under this system, *P. jiroveci* was called *P. carinii* formae specialis *hominis* (*P. carinii* f. sp. *hominis*). After these provisional nomenclature were instituted, more DNA sequence data were obtained, and by 2001, it became clear that the organism causing PCP in humans should be recognized as a distinct species. The name *P. jiroveci* had already been published in a valid manner in 1999 (15); however, publication of a name does not necessarily lead to its use. Therefore, at the 2001 International Workshop on Opportunistic Protists held in Cincinnati, Ohio, approximately 50 researchers from around the world, including clinicians, epidemiologists, and laboratory scientists, met to discuss the desirability and appropriateness of retaining the currently used trinomial nomenclature system, as opposed to assigning (or using) new species names. The group unanimously endorsed a proposal to rename the organisms currently known as special forms of *P. carinii* as species in the genus *Pneumocystis* and drew up guidelines for the creation of the new species names (16). Consequently, in keeping with the International Code of Botanical Nomenclature, it is no longer correct, either biologically or taxonomically, to refer to the human *Pneumocystis* organism as *P. carinii. P. carinii* now refers exclusively to the organism formerly known as *P. carinii* f. sp. *carinii,* one of the two *Pneumocystis* species found only in rats.

The consensus achieved at the workshop will help to make published reports on *Pneumocystis* more uniform with respect to nomenclature. Such uniformity will clarify communication among all who are interested in this genus and the disease caused by its members. Hopefully, all future reports pertaining to *P. jiroveci* will use its new name.

### Acronym "PCP" Retained

Given the compelling evidence that the human form of *Pneumocystis* is a separate species, the most important objection to designating it as such has been the problem that this name change could create in the medical literature, where the disease caused by *P. jiroveci* is widely known as PcP, or PCP. This problem can be avoided by taking the species name out of the disease name. Under this system, PCP would refer to *Pneumocystis* pneumonia. This simple modification in the vernacular accommodates the name change pertaining to the *Pneumocystis* species that infects humans. Furthermore, adopting this change makes the acronym appropriate for describing the disease in every host species, none of which, except rats, is infected by *P. carinii*.

### Multiple Strains of *P. Jiroveci*

DNA sequence polymorphisms are often observed in isolates of *P. jiroveci*, suggesting that numerous strains of this species exist. Loci that have been favorite targets for sequence analysis include the mitochondrial large subunit ribosomal RNA gene, the mitochondrial small subunit rRNA gene, the internal transcribed spacer regions of the nuclear rRNA gene (ITS), the *arom* gene, and the dihydropteroate synthase (DHPS) gene. The first three of these loci are considered to be under little if any selective pressure and presumably serve as indicators of genetic changes that are phenotypically neutral. The changes in the *arom* gene may also be considered neutral because they effect no change in the amino acid sequence of the enzyme. By contrast, the polymorphisms in the DHPS gene may be due to selection (see below). Techniques other than DNA sequencing have been used to detect genotypic variation. These include the use of type-specific oligonucleotide probes to detect variation at the ITS regions (40) and detection of single-strand conformation polymorphism (SSCP) at multiple loci (41).

 Genotyping has produced data from hundreds of *P. jiroveci* samples. Most studies have targeted one locus for analysis, but several multilocus studies have been reported (41–44). The allelic sequence polymorphism common in *P. jiroveci* is not seen in *P. carinii* (rat-derived *Pneumocystis*). However, *P. carinii* populations differ with respect to chromosome size, and several different strains have been identified by analysis of chromosome sizes (45,46). The possibility of chromosome size variation in *P. jiroveci* has not been adequately addressed because this analysis requires more organisms than are typically available from patients.

## New Perspectives on Infection

Genotyping samples of *P. jiroveci* provides a method for exploring epidemiologic issues. For example, one study examined the possibility that the low incidence of PCP in African HIV-infected persons might be due to the presence or absence of certain strains of *P. jiroveci*. However, samples of *P. jiroveci* from Zimbabwe, Brazil, the United States, and the United Kingdom have exhibited no major differences in genotypes (47,48). Another example is a study in which genotyping at four different genetic loci was used to compare isolates of *P. jiroveci* collected before (1968–1981) and after (1982 to present) the beginning of the AIDS pandemic (47). Pre- and postpandemic samples were the same except for a single base polymorphism (in the mitochondrial large subunit rRNA gene) found in the pre-pandemic samples only. These data show that the large increase in incidence of PCP was not accompanied by a shift in the kinds or frequencies of strains of *P. jiroveci.*

Strain analysis has also led to observations that are difficult to reconcile with the traditional view of the relationship between *P. jiroveci* and humans. The traditional theory holds that clinically important infection results from reactivation of a latent infection that was acquired during childhood. While infection of young children appears to be common, latent *P. jiroveci* has not been directly observed in healthy adults. In addition, indirect evidence is difficult to reconcile with lifelong latency.

The latency issue is important for several reasons. Under the reactivation of latent infection theory, little rationale exists for instituting measures to minimize the risk of infection during adulthood because this infection has already occurred. On the other hand, person-to-person transmission of the disease would have important public heath implications for medical centers that treat HIV-infected patients or other immunocompromised persons (42–44,49–52). Furthermore, transmission from patients who are undergoing treatment for PCP might enhance the opportunity for drug resistance to arise. By contrast, the generation of drug resistance would be less of a concern if most or all infections were due to transmission from an immunocompetent person, such as a young child's mother, or another child (i.e, someone who is not being treated for PCP). Under these conditions, drug-resistant strains, if they arose, would not spread very effectively.

PCP develops in infants infected with HIV perinatally, suggesting that *P. jiroveci* was present in these infants’ environments early in their lives (53). Evidence of *P. jiroveci* has also been found in some victims of sudden infant death syndrome (SIDS) (54). In normal, healthy children, serologic data have long indicated that infection of young children is common. Most children develop anti-*Pneumocystis* antibodies early in life, and the prevalence of these antibodies appears to increase with age (48,55). Recently, *P. jiroveci* has been linked to clinical illness in normal, healthy infants (51). *P. jiroveci* DNA was identified in nasopharyngeal aspirates obtained during episodes of mild respiratory infection in 24 (32%) of 74 infants. Seroconversion developed by 20 months of age in 67 (85%) of 79 infants who remained in the study and occurred in the absence of any symptoms of disease in 14 (18%). These reports confirm previous ones showing infection of children (1,3,4). Young children may be a reservoir of infectious *P. jiroveci* in the community.

Although infection of children seems common, little evidence exists for lifelong latency. Using PCR, Wakefield found no evidence of *P. jiroveci* in bronchoalveolar lavage fluid from 10 healthy persons (56). Peters replicated this result in postmortem lung tissue from 15 immunocompetent adults (56,57). (The techniques used to detect *P. jiroveci* have found it in HIV-negative adults but only those with other health problems [58].) Studies on recurrent PCP have shown that different *P. jiroveci* genotypes are present during different PCP episodes in patients with repeat episodes of PCP, a result suggestive of infection proximal to the time of disease (42-44). Recent infections of adults are also suggested by the high frequency of mutations that cause changes in the sequence of the DHPS gene, the enzyme associated with sulfonamide resistance in other pathogens (59–61). These mutations have not been detected in patients in whom PCP occurred at a time before the widespread use of sulfonamides to treat and prevent it (62) but are common in today's patients, even in those with no known exposure to sulfonamides (61,63). Mutant DHPS genes have been found in a variety of *P. jiroveci* genetic backgrounds, suggesting that selection for DHPS mutations is an ongoing process (64).

An alternative approach to exploring the importance of latency is employing population genetics and epidemiology to test the following hypothesis. If lifelong latency is important, adult patients who reside far from their birthplace should have the strain of *P. jiroveci* common in their place of birth, not in their place of residence. Data pertaining to this hypothesis are now available (64). The strains infecting adult patients were more similar to those common in their place of residence than their place of birth, suggesting that infections had been recently acquired, rather than carried since early childhood.

 Latent *P. jiroveci* have not been found in healthy adults, but proving that they do not exist is practically impossible. A single organism anywhere in the body could be sufficient to maintain a latent infection. Therefore, the possibility of latency remains. However, latent infections may be transitory, and humans who have eliminated the microbe may be subject to reinfection. The observations described above seem more consistent with this “transient colonization” scenario than with lifelong latency.

## Summary

 The microbe that causes PCP in humans is a distinct phylogenetic fungal species called *Pneumocystis jiroveci*. This species has been difficult to find in the environment, has not been found in nonhuman hosts, and is either absent in healthy humans or present at very low levels. In contrast, *P. jiroveci* is fairly common in humans who have depressed immune function. The number of *P. jiroveci* in a person appears to be dependent on the degree of immune dysfunction, suggesting that the species is adapted to exploit this dysfunction, growing to very high numbers in the severely immunodeficient and to lesser extents when immune function is less impaired. *P. jiroveci* may be eliminated when immune function is optimal. Genetic variants of the organism are common, providing markers for epidemiologic studies. Studies using these markers have raised questions about the role of latency in PCP. Recurrent PCP can be accompanied by shifts in genotype. Some patients are infected by genotypes more common in their place of residence than in their birthplace. Variable loci include the gene encoding an enzyme targeted by sulfonamides, suggesting transmission from treated patients to others at risk. While these observations, combined with the scarcity of *P. jiroveci* in healthy adults, do not exclude latency as a cause of PCP, they suggest that long-term latency is not the only source of this disease.
